# Novel Functional Features of cGMP Substrate Proteins IRAG1 and IRAG2

**DOI:** 10.3390/ijms24129837

**Published:** 2023-06-07

**Authors:** Sally Prüschenk, Michael Majer, Jens Schlossmann

**Affiliations:** Department of Pharmacology and Toxicology, Institute of Pharmacy, University of Regensburg, 93040 Regensburg, Germany; sally.prueschenk@chemie.uni-regensburg.de (S.P.); michael.majer@chemie.uni-regensburg.de (M.M.)

**Keywords:** cGMP, cGKI, IP_3_R-I, IP_3_R-II, IP_3_R-III, IRAG, IRAG1, IRAG2, Jaw1, LRMP, MRVI1, PKGI

## Abstract

The inositol triphosphate-associated proteins IRAG1 and IRAG2 are cGMP kinase substrate proteins that regulate intracellular Ca^2+^. Previously, IRAG1 was discovered as a 125 kDa membrane protein at the endoplasmic reticulum, which is associated with the intracellular Ca^2+^ channel IP_3_R-I and the PKGIβ and inhibits IP_3_R-I upon PKGIβ-mediated phosphorylation. IRAG2 is a 75 kDa membrane protein homolog of IRAG1 and was recently also determined as a PKGI substrate. Several (patho-)physiological functions of IRAG1 and IRAG2 were meanwhile elucidated in a variety of human and murine tissues, e.g., of IRAG1 in various smooth muscles, heart, platelets, and other blood cells, of IRAG2 in the pancreas, heart, platelets, and taste cells. Hence, lack of IRAG1 or IRAG2 leads to diverse phenotypes in these organs, e.g., smooth muscle and platelet disorders or secretory deficiency, respectively. This review aims to highlight the recent research regarding these two regulatory proteins to envision their molecular and (patho-)physiological tasks and to unravel their functional interplay as possible (patho-)physiological counterparts.

## 1. Introduction

The inositol 1,4,5-triphosphate receptor-associated cGMP kinase substrate 1 (IRAG1) and inositol 1,4,5-triphosphate receptor-associated 2 (IRAG2) are regulatory proteins that interact with the intracellular Ca^2+^ channels inositol trisphosphate receptors (IP_3_ receptors/IP_3_R) and thereby modulate their physiological functions. The IRAG1 protein represents a 125 kDa membrane protein located at the endoplasmic reticulum (ER) or sarcoplasmic reticulum (SR), respectively. IRAG1 was previously identified as a substrate protein, which interacts with cyclic guanosine monophosphate (cGMP) dependent protein kinase (PKG) Iβ isoform and regulates the IP_3_R-I function [1,2]. Then, it was elucidated that IRAG1 mediates the Ca^2+^-dependent functions of cGMP in a variety of tissues, e.g., in the vasculature, in the gastrointestinal system, and in platelets. These studies, performed with transgenic mice, showed that *Irag1* deficiency leads to gastrointestinal and vascular dysfunction [3,4] and arterial thrombosis [5]. Interestingly, IRAG1 is homologous to the putative tumor suppressor protein murine retrovirus integration site 1 (MRVI1), which is expressed in myeloid immune cells [6]. Hence, it might be possible that IRAG1 is also involved in immunological functions.

**Figure 1:**
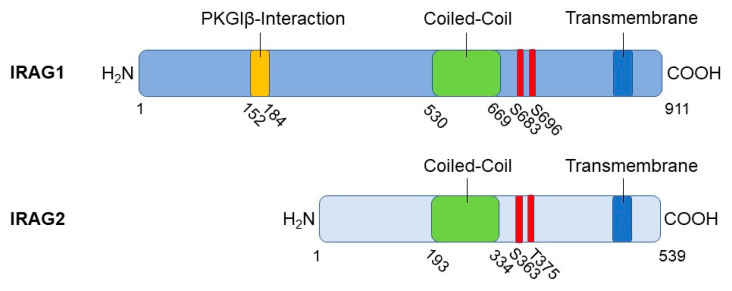
Schematical illustration of the primary structure and protein motifs of IRAG1 and IRAG2. Numbers indicate position of amino acids. PKGI phosphorylation sites are depicted in red (S: serine and T: threonine).

IRAG2, which is also called lymphoid-restricted membrane protein (LRMP) or Jaw1, is a 75 kDa ER membrane protein and is particularly homologous to IRAG1 in its coiled-coil region (Figure 1) [7,8,9]. This region is essential for the IP_3_R-I interaction of IRAG1 [1] and IRAG2 [8]. Jaw1/LRMP was previously identified in lymphoid cells [7]. There were indications that it might also be involved in the development of lymphoma [10] or type 1 diabetes [11,12]. However, its molecular function was not established in these previous publications. Further investigations located Jaw1/LRMP in taste cells, and it was thereby concluded that this protein might be associated with the perception of taste [8].

Recently, several new studies revealed new molecular details and several novel functions of IRAG1 and IRAG2. Therefore, this review aims to summarize these important findings and shall give new aspects for studies of these regulatory proteins in the future.

## 2. Functional Features of IRAG1

### 2.1. Structure, Interaction Partners, and Cellular Functions of IRAG1

The inositol 1,4,5-triphosphate receptor-associated cGMP kinase substrate 1 (IRAG1), also known as its human homolog murine retrovirus integration site 1 (MRVI1), is, like IRAG2, a type II membrane protein linked to the ER via a C-terminal hydrophobic region. This hydrophobic region acts as a membrane anchor (Figure 1) [2,6]. Two isoforms of IRAG1 exist, IRAG1a and IRAG1b, which differ in their amino acid sequence at the N-terminus, caused by alternative splicing. IRAG1a is the larger isoform due to 911 amino acids compared to IRAG1b with 859 amino acids [2,6]. For this reason, both molecules also differ in their molecular mass, which was calculated to be 90 kDa for IRAG1a and 98 kDa for IRAG1b [6]. However, the molecular weight for IRAG1a is 130 kDa, and for IRAG1b, it is 125 kDa, so an average molecular mass of 125 kDa can be assumed [2]. A high content of prolines and glycosylations is discussed as a possible cause for the actually larger molecular masses compared to the predicted molecular mass [6]. Studies on the human *IRAG1* gene revealed that splice variants of IRAG1 are truncated at the N- and C-terminal end. The C-terminal truncated variants act in a dominant-negative manner to counteract cGMP/PKGI signaling [13].

**Figure 2:**
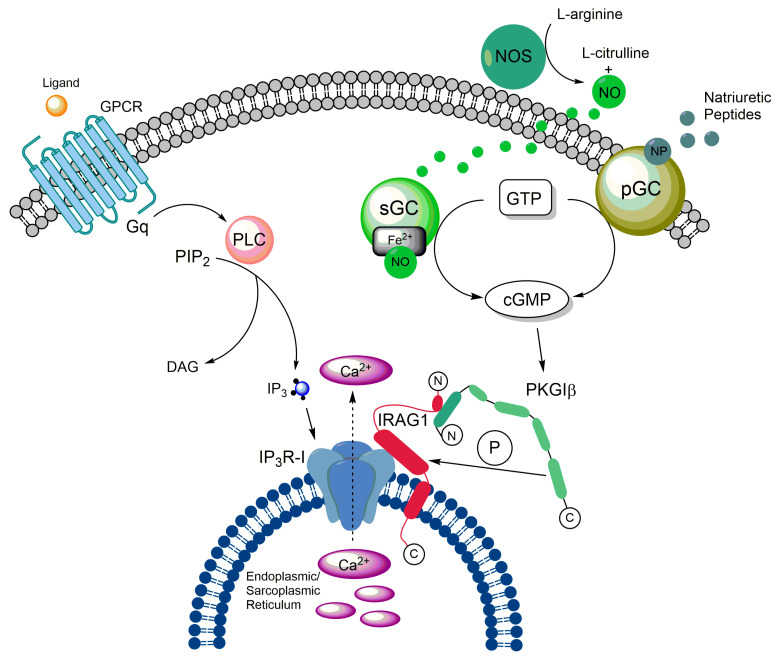
Graphical illustration of the PKGIβ/IRAG1 signaling pathway. The PKGIβ (light green) interacts with IRAG1 (red) by its leucine zipper region (dark green). After cGMP-dependent phosphorylation, IRAG1 inhibits Ca^2+^ release from the endoplasmic/sarcoplasmic reticulum (dashed arrow) via the IP_3_R-I (blue). For detailed description, see text below.

IRAG1 forms a macrocomplex with PKGIβ and IP_3_R-I, which is localized to the membrane of the ER to regulate the release of Ca^2+^ from this cellular structure via IP_3_R-I [1,2]. To this end, IRAG1 exhibits several structural features in addition to the membrane anchor already mentioned. For protein–protein interactions, it has a coiled-coil domain with a phosphorylation site for PKGIβ at each of its C- and N-terminal ends [1,2,14]. Via amino acids 152 to 184, bovine IRAG1 interacts with the leucine zipper of PKGIβ located at its N-terminus but not with its linker region (Figure 1). By comparing the amino acids involved in the interaction between the two proteins, it can be assumed that the interaction occurs via electrostatic interactions [1]. Based on the following studies, this assumption could be confirmed by showing that acidic residues in the leucine zipper of PKGIβ and small clusters of basic residues of IRAG1 are involved [14]. The interaction results in the phosphorylation of bovine IRAG1 at serine (Ser) 696 (Figure 1) and at Ser677 of human IRAG1, respectively, and inhibits the release of Ca^2+^ mediated via IP_3_R-I [1,5]. Furthermore, the interaction between IRAG1 and PKGIβ ensures that the PKGIβ reaches the ER, limiting its nuclear translocation and inhibiting its ability to mediate transcription [15]. This translocation could not be observed in vascular smooth muscle cells (VSMCs) of global *Irag1*-deficient mice [4]. However, the loss of the interaction between IRAG1 and PKGIβ may be the reason why protein expression of PKGIβ is reduced in *Irag1* mouse mutants [3,4,16,17]. In contrast, the coiled-coil domain of IRAG1 is not involved in any interaction between IRAG1 and PKGIβ, but is necessary for the interaction of IRAG1 with the IP_3_R-I [1]. Furthermore, no protein interactions of IRAG1 with PKGIα and PKGII were observed [1,2,14]. Deletion of exon 12, which encodes the N-terminus of the coiled-coil domain, resulted in a 5 kDa smaller IRAG1 protein, thus causing the interruption of the interaction with IP_3_R-I in COS-7 cells and murine smooth muscle tissue. In this context, it was further shown that the PKGIβ/IRAG1 interaction with IP_3_R-I is crucial for cGMP-dependent smooth muscle cell relaxation controlled by receptors (Section 2.4) [3]. A graphical illustration of the PKGIβ/IRAG1 signaling pathway is shown in the following figure (Figure 2).

New results showed that IRAG1 is an interaction partner of hyperpolarization-activated cyclic nucleotide-sensitive isoform 4 (HCN4) channels. Expression of HCN4 and IRAG1 in HEK293 cells, followed by immunoprecipitation, resulted in IRAG1 as an interaction partner of HCN4. As an isoform-specific modulator of this channel, IRAG1 increases its activity to more depolarized potentials in the absence of cyclic adenosine monophosphate (cAMP) [18].

### 2.2. Expression Pattern and Localization of IRAG1

IRAG1 is a widely expressed protein in mammalian tissues. In the first description of MRVI1, RNA expression of the gene was detected in various human and murine tissues, e.g., heart, brain, and skeletal muscle, as well as myeloid cells [6]. Northern blot data of IRAG1 showed the same result in human tissues as when first described [2]. Further analysis of the expression and localization revealed that IRAG1 was detectable in a variety of murine tissues. High amounts were found in smooth muscle-containing tissues such as the aorta, stomach, or colon and in platelets. Only small amounts were found in the heart or the spleen [19]. In this context, the expression and localization of PKGIβ, which is one of the interaction partners of IRAG1, were also investigated [2]. The expression and tissue distribution of PKGIβ correlated with that of IRAG1. In the brain, this correlation was not associated with the localization of both proteins [19]. Cellular localization studies exposed that heterologously expressed IRAG1 was detectable perinuclearly, which supports the idea that IRAG1 is located in the membrane of the ER, where it forms a macrocomplex with the PKGIβ and IP_3_R-I [2,6,19]. In freshly isolated smooth muscle cells (SMCs) of cerebral arteries, the macrocomplex, consisting of IRAG1, PKGIβ, and IP_3_R-I, was localized to the SR, which is the equivalent of the ER in muscle cells [20].

### 2.3. Impact of IRAG1 on Ca^2+^ Signaling

The formation of the trimeric complex of PKGIβ, IP_3_R-I, and IRAG1 raised the question of the importance of IRAG1 for the release of Ca^2+^. Thereby, it could be shown in COS-7 cells that IP_3_R-I mediated Ca^2+^ release is suppressed in dependence on cGMP after stimulation with bradykinin, as soon as PKGIβ and IRAG1 are coexpressed in these cells. Similarly, IRAG1 inhibits the IP_3_-induced release of Ca^2+^ in a cGMP-dependent manner [2]. This regulation of Ca^2+^ release is achieved by phosphorylation of IRAG1 due to PKGIβ at Ser696 of bovine IRAG1 [1]. To investigate the influence of IRAG1 on Ca^2+^ release under physiological conditions, experiments such as those performed in the COS-7 expression system were conducted in cells of the human colon. Again, IRAG1 was shown to inhibit the cGMP-dependent release of Ca^2+^ from the ER-mediated by IP_3_R-I, and seems to be involved in the control of nitric oxide (NO)-dependent relaxation of gastrointestinal muscles [21]. Measurements with aortic smooth muscle cells gave comparable results. Therefore, the cGMP-dependent release of Ca^2+^ was not reduced in the VSMCs of *Irag1*^Δ12/Δ12^ mouse mutants compared to those of wild-type mice. This result supports the fact that the interaction between IRAG1 and the IP_3_R-I is essential for cGMP-dependent regulation of Ca^2+^ release (Figure 2) [3]. Studies on IP_3_ receptors confirmed the inhibitory function of IRAG1 on Ca^2+^ release due to IP_3_R-I, whereas the absence of IRAG1 resulted in enhanced Ca^2+^ release. Interestingly, IRAG1 was also observed to inhibit the direct modulation of IP_3_R-I by protein kinase A (PKA) and the PKA-dependent phosphorylation of IP_3_R-I [22].

In summary, IRAG1 is of critical importance in the regulation of Ca^2+^ release from the ER via IP_3_R-I.

### 2.4. (Patho-)Physiological Functions of IRAG1

#### 2.4.1. IRAG1 and the Gastrointestinal System

PKGI is of important relevance to the function of gastrointestinal SMCs [23]. The fact that IRAG1 forms a complex together with the PKGIβ and is phosphorylated by this enzyme combined with the strong expression of IRAG1 in the gastrointestinal tract suggests that IRAG1 is also of vital importance there [1,2,19,21]. Therefore, the (patho-)physiological function of IRAG1 was investigated in more detail by mouse models: the *Irag1*^Δ12/Δ12^ mouse and the global *Irag1*-deficient (*Irag1*^-/-^) mouse [3,4]. *Irag1*^Δ12/Δ12^ mice reveal an interruption of the interaction between IRAG1 and IP_3_R-I, caused by the deletion of exon 12 of *Irag1*, which encodes the N-terminal part of the coiled-coil domain. The loss of the interaction between IRAG1 and IP_3_R-I did not affect the carbachol-induced contraction but impaired the cGMP-mediated relaxation of SMCs of the colon as well as the tonic phase of depolarization-induced contraction [3,24]. Similar results were found in colonic SMCs of global *Irag1*-deficient mice [4]. However, in the jejunum of *Irag1*^Δ12/Δ12^ mice, this was not observed [24]. This confirmed the important function of IRAG1 in the cGMP-mediated relaxation of receptor-triggered contraction of SMCs. Furthermore, these results approved the interaction between IRAG1 and the IP_3_R-I and showed that PKGIβ/IRAG1 signaling is of major importance for the cGMP-mediated relaxation of smooth muscles.

Studies on the physiological functions of IRAG1 in mouse models showed that IRAG1 is crucial for the functionality of the gastrointestinal tract in toto. *Irag1*^Δ12/Δ12^ mice developed an enlarged gastrointestinal tract, impaired gastrointestinal motility, and pyloric stenosis [3]. These pathological changes could also be observed in global *Irag1*^-/-^ mice [4]. Further studies with these transgenic mice revealed that they had iron deficiency anemia as a result of gastrointestinal bleeding and subsequently developed splenomegaly [16]. The results verified indications of splenomegaly in earlier studies of *Irag1* mouse mutants [3,4]. Interestingly, these findings occurred mainly in female *Irag1*-deficient mice [16]. In this context, it was shown that IRAG1 has a function in the development of achalasia. Achalasia is a disease of the gastrointestinal tract that leads to impaired esophageal motility and incomplete relaxation of the lower esophageal sphincter. In two achalasia patients, a homozygous nonsense mutation of *IRAG1* was detected. This mutation resulted in a loss of the known interaction of IRAG1 and PKGIβ. As a consequence, the central role of IRAG1 in the regulation of Ca^2+^ levels and the associated regulation of cGMP-regulated smooth muscle relaxation was lost [25]. This case report is consistent with previously reported data showing that microRNA (miRNA) regulates *IRAG1* in esophageal SMCs in achalasia patients [26]. In *Irag1* mouse mutants, it was further observed that mutation or deficiency of IRAG1 caused a decrease in protein expression of PKGIβ [3,4,16]. The reduced protein expression of PKGIβ could not be explained by a decrease in the corresponding gene expression [16]. In this context, it is interesting to know that *Prkg1* mouse mutants—which do not encode both isoforms of the PKGI—develop a similar phenotype to the *Irag1* mouse mutants, only in a much stronger manifestation [27,28,29].

Combining the known data on IRAG1 and PKGIβ suggests that a disruption of the PKGIβ/IRAG1 signaling pathway can cause the development of gastrointestinal disorders.

#### 2.4.2. IRAG1 and (Cardio-)Vascular System

Besides the gastrointestinal SMCs, IRAG1 is also highly expressed in VSMCs and the heart [19]. In vitro experiments with VSMCs of *Irag1*^Δ12/Δ12^ and *Irag1*^-/-^ mice gave comparable results to those in the SMCs of the colon. The loss of the interaction between IRAG1 and IP_3_R-I impaired cGMP-mediated relaxation of VSMCs of the aorta [3,4]. Furthermore, in global *Irag1*-deficient mice, the cGMP- and atrial natriuretic peptide (ANP)-mediated relaxation of VSMCs was reduced after acetylcholine-induced contraction. Thus, IRAG1 has a central role in the NO/cGMP- and ANP-mediated relaxation of VSMCs [4]. The detailed mechanism of cGMP-mediated relaxation of VSMCs has not yet been conclusively determined. A possible mechanism could be an interaction between IRAG1 and the transient receptor potential melastatin 4 channel (TRPM4). These sodium channels are activated by Ca^2+^ from the SR and take part in the vasoconstriction of blood vessels. In cerebral arteries, IRAG1 and TRPM4 were colocalized in the SR of those VSMCs. When targeting *Irag1* with morpholinos, the NO-induced vasodilation was blunted, and the activity of TRPM4 was reduced. This indicates that NO/cGMP/PKG signaling decreases TRPM4 activity through an IRAG1-mediated inhibition of Ca^2+^ release from the SR [20]. Moreover, it was shown that not only cGMP promotes relaxation in VSMCs via IRAG1. The cyclic nucleotide cyclic cytidine 3′,5′-monophosphate (cCMP) also influences their relaxation. So, it was demonstrated that cCMP-mediated relaxation is also reduced in *Irag1*-deficient VSMCs in analogy to cGMP-mediated relaxation. It can be assumed that the effects of cCMP occur via the stimulation of PGKI, which then leads to the phosphorylation of IRAG1 [30]. Physiological investigation of IRAG1 in *Irag1*-deficient mice exposed that a loss of IRAG1 caused mild pulmonary arterial remodeling and an increasing percentage of muscularized arteries if these mice were kept under normoxic conditions [17]. A genetic analysis of a Caucasian family with moyamoya syndrome (MMS), which was associated with neurofibromatosis type 1 (NF1), revealed a polymorphism of *IRAG1* in exon 5. This polymorphism might represent *IRAG1* as a genetic susceptibility factor for MMS in NF1 [31]. Furthermore, *IRAG1* was one of seven newly identified genes in the context of lacunar stroke and was abundantly expressed in astrocytes [32].

Based on the physiological function of IRAG1 in VSMCs, the role of IRAG1 in the heart was of interest as it is also expressed there [6,17,18,19]. A good parameter to assess this is the heart function. *Irag1* mouse mutants have a slight tendency for hypotension [3,4]. However, after the induction of sepsis, blood pressure remained constant in *Irag1*-deficient mice in contrast to wild-type mice [4]. As already mentioned, *Irag1*^-/-^ mice did not show any difference in blood pressure. Though, under normoxic conditions, an *Irag1* deficiency caused a significant increase in the systolic pressure of the right ventricle (RV), and they develop RV hypertrophy and dilatation. However, no evidence of heart fibrosis was recognized. Thus, an *Irag1* deficiency causes spontaneous development of pulmonary hypertonia (PH) under normoxic conditions without any trigger, e.g., hypoxia [17]. This finding is consistent with the data on pulmonary vascular remodeling of *Irag1*^-/-^ mice, as pulmonary vascular remodeling is often associated with PH. The cardioprotective function of IRAG1 is also supported by other results. Burn-induced cardiomyopathy in rats causes a decrease in cGMP and various genes, such as *Irag1* and *Prkg*. If these rats were treated with the phosphodiesterase (PDE) 5 inhibitor sildenafil, there was a normalization of cardiac function and an increase in cGMP and mRNA levels, among others of *Irag1* and *Prkg*. This indicates that PDE5/cGMP/PKG are mediating burn-induced heart dysfunction [33]. In the process of demonstrating HCN4 as a novel interaction partner of IRAG1, it was detected in the sinoatrial node of murine hearts. Based on a model of funny current (I_f_), it was predicted that IRAG1 increases the I_f_ in sinoatrial myocytes [18]. So, IRAG1, as an HCN4 modulator, is suggested to have a possible role in heart rate regulation [34].

Furthermore, IRAG1 is expressed in the lungs [19]. Analysis of the expression of IRAG1 protein in lungs and isolated pulmonary artery of smooth muscle cells (PASMCs) of end-stage idiopathic pulmonary arterial hypertension (IPAH) revealed an increased expression of IRAG1 and PKGIβ. This contrasted with hypoxic *Irag1*-deficient mice. They showed a decreased PKGIβ protein expression in lung and murine PASMCs [17].

IRAG1 is not only expressed in tissues of the cardiovascular system, but also in a huge amount in platelets [19]. The facts that PKGI has a crucial function in platelet aggregation and that IRAG1 is a substrate of the PKGIβ and also forms a macrocomplex with PKGIβ and IP_3_R-I raised the question of the physiological function of IRAG1 in platelets [2,35]. This well-known macrocomplex, as well as the phosphorylation of IRAG1 by PKGIβ, which results in the inhibition of Ca^2+^ release from IP_3_R-I, was found in human and murine platelets. However, in human platelets, two phosphorylated serine residues were identified—Ser664 and Ser677—whereby the Ser677 in human IRAG1 corresponds to the Ser696 in bovine IRAG1 [2,5,36]. The examination of platelets from *Irag1*^∆12/∆12^ and global *Irag1*-deficient mouse mutants revealed that cGMP- and NO-induced platelet aggregation was inhibited in these mouse mutants [5,36]. cAMP- or prostacyclin-mediated aggregation was not affected, and the cGMP- or NO-mediated and thrombin-induced Ca^2+^ release was not suppressed in the mutant platelets [5]. Therefore, IRAG1 has a crucial function in impeding NO/cGMP signaling in platelet aggregation by suppression of intracellular Ca^2+^ [5]. Additionally, there might be an indirect effect of the PKGIβ/IRAG1/IP_3_R-I macrocomplex mediating compartmentation and, thereby, regulation of PDE5, e.g., in platelets [37]. However, not only the cGMP-mediated inhibition of platelet aggregation is affected by IRAG1. The cCMP-mediated inhibition of aggregation was also inhibited in *Irag1*-deficient platelets. This suggests that similar to VSMCs, cCMP-regulated effects are operated by the PGKI/IRAG1 pathway [30]. In addition, IRAG1 is involved in the NO- or cGMP-induced inhibition of adenosine triphosphate and serotonin secretion from dense granules and P-selectin secretion from alpha granules in platelets [36]. The circumstance that global *Irag1*-deficient mice showed reduced bleeding time confirmed the results on the physiological function of IRAG1 determined in in vitro experiments [36]. Furthermore, IRAG1 is necessary for the cGMP-dependent inhibition of platelet activation and prevention of arterial thrombosis as well as for the inhibition of thrombin-induced adhesion of platelets to fibrinogen [5,36]. These experimental data on the physiological function of IRAG1 are supported by several clinical data. Meta-analyses discovered single nucleotide polymorphisms (SNPs) in the human *IRAG1* gene, which cause an increased aggregability of platelets to agonists and reduce mean platelet volume [38,39,40].

Taken together, IRAG1 is an important protein for the physiological function of the (cardio-)vascular system and for platelet function. These findings and the participation in the development of disorders make IRAG1 an interesting target for further studies and possible pharmacological treatments.

#### 2.4.3. IRAG1 and Cancer

In addition to the (patho-)physiological functions of IRAG1 described so far, it is also important in tumor diseases. This was already evident in the first description of *IRAG1*, respectively *MRVI1*, where it was detected in BXH2 leukemias. It was concluded that *IRAG1* can induce myeloid leukemia by altering the expression of a gene important for myeloid cell growth and/or differentiation, and it was suspected that *IRAG1* may function as a tumor suppressor gene [6]. Involvement of IRAG1 in myeloid leukemias was also demonstrated in another study investigating the extent to which the recurrent chromosomal translocations of the tyrosine kinases BCR-ABL, TEL-PDGFRB, and TEL-JAK2 regulate distinct and overlapping gene transcription profiles. It was shown that *IRAG1* was increasingly expressed in Ba/F3 cells after transfection with either BCR-ABL or TEL-PDGFRB. Again, it was concluded that *IRAG1* plays a significant role in the development of leukemia. However, this remains to be conclusively elucidated [41]. This statement is supported by the findings that CD300A was upregulated in patients with acute myeloid leukemia of the intermediate or adverse risk category of the WHO criteria (2018) and predicts poor survival. CD300A upregulation stimulates the cGMP/PKG signaling pathway, and *IRAG1* was positively correlated with CD300A [42].

Besides its involvement in the development of myeloid leukemia, IRAG1 is also important in solid tumors. The association of IRAG1 with tumors of the female reproductive system has been described so far. The response of patients suffering from serous ovarian carcinoma stage III to cytostatic therapies varies. It was reported that *IRAG1* was upregulated in 61% of these patients. Overexpression of *IRAG1* has a direct impact on survival because these patients have a significantly worse prognosis in terms of survival than those in whom *IRAG1* was not upregulated. Furthermore, if the tumor was chemo-resistant, the prognosis was even worse. These results implicate that *IRAG1* is involved in the chemo-resistance of serous ovarian carcinoma [43]. While the involvement of IRAG1 in ovarian carcinoma is associated with a poor prognosis, this does not apply to endometrial carcinoma. MicroRNAs modulate cellular processes, and there is growing evidence that they are linked to the progression of diverse cancers, such as endometrial carcinoma. MicroRNA miR-940 acts as an oncogene during progression, and high expression is associated with, among other things, reduced overall survival. In these tumors, it was found that the expression of IRAG1, which is a direct target of miR-940, was decreased. Moreover, IRAG1 expression was associated with, e.g., survival of the patients in the context that high expression levels of IRAG1 were linked to a good prognosis. Thus, miR-940 regulates the progression of endometrial carcinoma by affecting the expression of IRAG1 [44]. Like endometrial carcinoma, low expression of *IRAG1* in cervical carcinoma was associated with poor overall survival. This is caused by hypermethylation in the promotor regions of the *IRAG1* promoter, resulting in low gene expression of *IRAG1* [45]. Furthermore, the gene expression of *IRAG1* was upregulated in the context of pancreatic ductal adenocarcinoma (PDAC) after silencing the transcription factor basic transcription factor 3 (BTF3) in pancreatic cancer cell lines [46]. Additionally, expression of *IRAG1* was negatively associated with high expression of the cell division cycle-associated protein 2 (CDCA2) in glioma [47].

Based on the current data, it can be concluded that it is not yet possible to make a clear statement on the function of IRAG1 in the development and/or progression of tumors. This is due to the fact that the data collected so far are established by bioinformatic analyses and investigations on corresponding cell lines. However, from these previous studies, it can be assumed that IRAG1 has different functions depending on the tumor type. The exact role of IRAG1 in these tumors remains to be investigated in systematic experimental work.

#### 2.4.4. Further (Patho-)Physiological Functions of IRAG1

Apart from the previously mentioned (patho-)physiological functions, IRAG1 might also have more functions. For example, an increased expression of *IRAG1* in addition to two other genes was described in keratoconus, but the role of *IRAG1* in this eye disease is still unclear so far [48]. The attachment and motility of osteoclasts are regulated by NO and PGKI, which modulates the release of Ca^2+^ by the IP_3_R-I. Functional studies on osteoclasts revealed that IRAG1 is expressed in these cell types and is required for the Ca^2+^ release during motility. If the IRAG1-mediated Ca^2+^ release from the IP_3_R-I was disrupted, this might be a cause for the dysfunction of osteoclasts [49]. IRAG1 reveals not only a significant role in the development of tumors of the female reproductive system, but it is also involved in other diseases in this body region. Proteomic analysis of tissue from intrauterine adhesions (IUA)—also known as Asherman’s syndrome—revealed IRAG1 as one of seven proteins that were upregulated in IUA. However, the exact mechanisms and signaling pathways remain to be investigated before IRAG1 can act as a potential target protein for the clinical treatment of IUA [50]. Further investigations showed that IRAG1 participates in endothelial ANP/cGMP/PKGI signaling [51].

Taken together, IRAG1 is of importance for several physiological functions due to its large tissue distribution, but this needs to be further investigated.

### 2.5. Polymorphisms of IRAG1 Gene

In recent years, several single nucleotide polymorphisms (SNPs) and variations (SNVs) were discovered in conjunction with *IRAG1*. *IRAG1* was identified as one of four loci that are associated with arterial stiffness index. However, in the following secondary analysis of these data, *IRAG1* was not genome-wide significant, but it represented an interesting candidate [52]. SNPs of *IRAG1* were also identified in the context of migraines. There were associations of *IRAG1* with migraines, and combined with cervical artery dissection (CeAD), there were associations for CeAD mapped to the *IRAG1* gene [53,54]. As described in Section 2.4.2, polymorphisms of the *IRAG1* gene were associated with NF1-associated MMS [31]. Platelet aggregation was also affected by variations of the *IRAG1* gene. They caused an increased aggregability of the platelets to agonists [38,39,40]. Nevertheless, not all polymorphisms of *IRAG1* were relevant for the development of dysfunctions. Patients with unstable angina, which had *IRAG1* polymorphisms, did not show any association between the polymorphism and the disease [55]. Based on data from the UK Biobank study, there was an association between SNP of *IRAG1* and childhood-onset asthma [56].

Thus, genetic variations of *IRAG1* have a significant role in cardiovascular disorders. However, their exact function and significance in the development and progression of the respective disease still need to be investigated in further experimental studies with the aim of clarifying the potential role of *IRAG1* in these processes and if it is suitable as a possible therapeutic target.

### 2.6. Significance of IRAG1 as a Diagnostical/Prognostic Marker

Independent of the known (patho-)physiological functions of IRAG1, it was shown to be important in the context of diagnosis and prognosis of diseases. However, the extent to which IRAG1 is suitable as a marker is still unclear or under investigation. For example, *IRAG1* was demonstrated to play a significant role in the prognosis of idiopathic pulmonary fibrosis (IPF). This is a chronic respiratory disease characterized by the peripheral distribution of bilateral pulmonary fibrosis, which is associated with a poor prognosis and short survival. Bronchoalveolar lavage cells from the Gene Expression Omnibus (GEO) database were evaluated, and *IRAG1* was identified as one of the relevant genes to the prognosis of IPF, among other genes [57,58].

As described in the previous section, expression of IRAG1 is associated with the prognosis of survival depending on the respective types of tumors (Section 2.4.3). It should be noted that these data are evaluations of databases, which should be further investigated to verify the previous results. However, it must be mentioned that cervical cancer expression and methylation levels of *IRAG1* could be effectively differentiated between cancer and healthy tissue samples [45].

Based on the present knowledge about the (patho-)physiological function of IRAG1, the currently available data indicate that IRAG1 could also be used as a diagnostic and/or prognostic marker in some fibrotic or cancer diseases. Furthermore, it can be assumed that during further research, IRAG1 may be used as a marker for diagnosis or progression in additional diseases.

## 3. Functional Features of IRAG2

### 3.1. Structure of IRAG2

The inositol 1,4,5-triphosphate receptor associated 2 (IRAG2) is also known as Jaw1 or lymphoid-restricted membrane protein (LRMP) and was first described in 1994 by Behrens et al. [7]. IRAG2 is a membrane protein consisting of 539 amino acids and is targeted to the cytoplasmic face of the ER [7,59]. Moreover, the localization of IRAG2 was shown at the outer nuclear membrane [60,61]. The insertion into the ER membrane occurs post-translationally [7,59]. After targeting the ER, IRAG2 is cleaved at its C-terminus, leading to the existence of a shorter IRAG2 fragment beside the full-length IRAG2 [59,60,62]. This cleavage event occurs at the C-terminal luminal domain between amino acid Ala509 and Ala510, and is achieved through the signal peptidase complex isoform SEC11A [62].

IRAG2 consists of a C-terminal anchor domain, which is important for its localization to the ER, a coiled-coiled domain, which is significant for protein–protein interactions, and an N-terminal domain with a cytoplasmic orientation (Figure 1). Due to its C-terminal hydrophobic anchor and the cytosolic N-terminal domain, IRAG2 is classified as a type II membrane protein [7]. In the short luminal domain of IRAG2 resides 39 carboxyl (C)-terminal amino acids, and it is important for the targeting of IRAG2 into the ER membrane. Replacement of the luminal domain through the luminal domain of other Klarsicht/ANC-1/Syne/homology (KASH) proteins leads to the localization of IRAG2 at the outer nuclear membrane but not at the ER [62].

Especially in its coiled-coil domain, IRAG2 reveals a homology of 44% to the coiled-coil domain of IRAG1 [6,8,18]. Therefore, IRAG1 and IRAG2 share some similar interaction partners. Like IRAG1, IRAG2 interacts with the IP_3_ receptors in a variety of tissues and cell-lines. Besides the interaction with the IP_3_ receptors, IRAG2—like IRAG1—interacts with the HCN4 channel in CHO cells and in HEK cells [18,34]. The interaction with these ion channels and receptors suggests a function of IRAG2 in the gating mechanisms of these channels and, therefore, an impact on physiological processes. Moreover, it was shown that IRAG2 interacts with Sad-1/UNC-84 (SUN) proteins and the microtubules [60].

### 3.2. Expression Pattern and Localization of IRAG2

First, localization studies of IRAG2 showed mRNA expression in lymphoid tissues and cell lines, such as B-cell lines and T-cell lines, where expression is regulated developmentally. The highest level of mRNA was found in pre-B-cells, pre-T-cells, and mature B-cells. Only low expression was detected in mature T-cells and plasma B-cells, suggesting a role of IRAG2 in lymphoid development. Furthermore, protein expression of IRAG2 was seen in the spleen and thymus. However, in nonhematopoietic cell lines and tissues, no expression of IRAG2 was reported in these first studies [7]. Later, Tedoldi et al. performed immunohistology investigations, where further expression of IRAG2 was shown in peripheral lymphoid tissues, such as lymph nodes and the tonsils. Thereby, high expression was detected in germinal center B-cells and monocytic B-cells but not in mantle zone B-cells and the interfollicular area. Furthermore, cortical thymocytes and splenic marginal zone cells were stained positive for IRAG2. In a bone marrow trephine, IRAG2 was detected in clustered normoblasts. This immunohistology analysis of IRAG2 also focused on hematolymphoid neoplasia-like lymphomas, where IRAG2 expression was detectable in B-cell lymphomas but not in T-cell neoplasms. Additionally, IRAG2 is expressed in lymphomas arising from germinal centers, Burkitt’s lymphoma, and lymphocyte-predominant Hodgkin’s disease. Hence, the expression in lymphoid neoplasms reflects the expression pattern that is observed in normal lymphoid tissues. Further, IRAG2 is detectable in almost all chronic lymphatic leukemias but not in classical Hodgkin’s disease. Besides the expression in lymphoid tissues, IRAG2 can also be found in non-lymphoid tissues and cells, such as neuronal cells in the cerebral cortex, epithelial cells in the tonsils and seminal vesicles, adrenal glands, and zymogen-producing cells in the stomach [10]. Subsequent studies also showed the presence of IRAG2 in various other tissues, such as sweet, bitter, and umami taste-responsive cells, where it might play a role in taste signal transduction [8]. Furthermore, expression of IRAG2 is seen in sinoatrial nodes [18] and in intestinal tuft cells [63], where it might exert important physiological functions due to its interaction with different ion channels or receptors. We recently detected the expression of IRAG2 in platelets and pancreatic acinar cells [9,64]. In these cells, IRAG2 engages in Ca^2+^ signaling, platelet aggregation, and enzymatic secretion (Section 3.4, Section 3.6.4 and Section 3.6.5).

### 3.3. Cellular Functions of IRAG2

The C-terminus of IRAG2 shows a partial homology with the PPPX motif of KASH proteins. This motif consists of four amino acids and is highly conserved between the five KASH proteins Nesprin1–4 and KASH5 [60,61]. Like IRAG2, KASH proteins also belong to the family of type II membrane proteins and are localized to the outer nuclear membrane. By its C-terminal luminal domain, KASH proteins interact with SUN proteins, which are localized to the inner nuclear membrane. This complex, called linker of nucleus and cytoskeletons (LINC), is formed in the perinuclear space by the interaction of a trimeric KASH protein with trimeric SUN proteins, resulting in a hetero-hexamer complex, which is necessary for maintaining the nuclear shape. Furthermore, KASH proteins interact with microtubules through their cytosolic region [65,66,67,68]. This interaction allows a connection of the cytoplasm to the nucleoplasm and is important for maintaining the shape and position of the nucleus [65,69]. For IRAG2, an interaction with SUN proteins and microtubules is also detected [60]. These findings suggest that IRAG2 acts as a KASH protein and is involved in maintaining the nuclear shape [60]. In a myeloma cell line, it is shown that depletion of IRAG2 leads to an aberrant nuclear shape, supporting this thesis. In conclusion, IRAG2 might function as part of the LINC-complex by interacting with microtubules on the cytosolic face and with SUN proteins in the perinuclear space via its KASH domain [60,61]. Furthermore, IRAG2 seems to act in an oligomeric state in this complex [60]. This oligomerization is probably achieved via the coiled-coil domain on the cytosolic face of the outer nuclear membrane [70].

Further studies showed that the N-terminal region of IRAG2 inhibits the formation of a smooth endoplasmic reticulum (OSER) [70]. It is known that overexpression and uncontrolled oligomerization of several ER-resident proteins can cause OSER formation, which is characterized by highly dense structures such as cisternae, nuclear karmellae, and whorls [70,71,72,73]. Kozono et al. reported that deletion of the N-terminal region of IRAG2 leads to its aberrant oligomerization and, consequently, to the formation of nuclear karmellae, where the ER membranes are stacked along the nuclear envelope. This phenomenon was observed for human and murine IRAG2, despite their relatively low percentage identity of the N-terminal region (43.6%). However, a computational analysis indicates that the N-terminal regions of both murine and human IRAG2 are intrinsically disordered regions (IDR) [70]. These regions are characterized by higher polarity and lower hydrophobicity compared to other structured regions, such as coiled-coil domains or transmembrane domains, resulting in an unstable conformational state [74,75]. Moreover, IDRs regulate protein–protein interactions and are involved in the regulation of precise oligomerization [75,76,77]. Hence, it is suggested that the N-terminal region of IRAG2 regulates the oligomeric state of IRAG2 as an IDR by preventing the structural exposure of the coiled-coil as an oligomerization site. Loss of the N-terminal domain, however, leads to aberrant oligomerization of the protein. Thus, the N-terminal region of IRAG2 prevents the formation of an OSER, which is essential for maintaining the homeostatic localization of an IRAG2 oligomer and its interacting partners on the ER membrane [70].

Several components of the LINC-complex are involved in the morphology and position of the Golgi apparatus [78,79,80]. As IRAG2 functions as a KASH protein to maintain nuclear shape, it might play a role in the formation of the Golgi apparatus [60,81]. Investigations using B16F10-cells showed that IRAG2 participates in maintaining the Golgi ribbon structure associated with the microtubule network. Knockdown of IRAG2 in these cells causes fragmentation of the Golgi apparatus and a loss of the Golgi ribbon structure. Furthermore, the depletion of IRAG2 disturbs the localization of the Golgi-derived microtubule network, indicating that IRAG2 keeps the compact structure of the microtubule-derived network and is needed to maintain the Golgi morphology. However, the mechanism of how IRAG2 affects the maintenance of the Golgi apparatus still needs to be clarified [81].

Moreover, these investigations of Okumura et al. indicate that IRAG2 might be associated with the position of the centrosome. As the correct position of the centrosome and Golgi apparatus is important for protein secretion, it is conceivable that IRAG2 has an impact on physiological functions derived from positional and morphological maintenance of the Golgi apparatus and the centrosome [81].

### 3.4. Impact of IRAG2 on Ca^2+^ Signaling

IP_3_ receptors are important Ca^2+^ channels, which are activated upon binding of IP_3_, leading to the release of Ca^2+^ from intracellular stores [82]. An interaction of IRAG2 with IP_3_R-III was shown for the first time in the COS-7-heterologous expression system, where IRAG2 was co-transfected with the IP_3_R-III [8]. Later, an interaction of IRAG2 was also detected with the IP_3_R-II in mouse embryo fibroblast (MEF) cells, and with IP_3_R-I, IP_3_R-II, and IP_3_R-III in HEK cells that were transfected with IRAG2 and one IP_3_ receptor subtype each [63,83]. Furthermore, we recently reported the interaction of IRAG2 with all IP_3_ receptor subtypes in the murine pancreas as well as in murine platelets [9,64]. The interaction with these receptors occurs through the coiled-coil domain of IRAG2 and impacts the release of Ca^2+^ from intracellular stores [8,83].

In MEF cells, which were co-transfected with IP_3_R-II and IRAG2, it is shown that IRAG2 enhances Ca^2+^ flux compared to MEF cells not expressing IRAG2 [63]. Furthermore, Okumura et al. reported an increased release of Ca^2+^ in HEK cells, which express IRAG2, compared to HEK cells lacking IRAG2. Moreover, mutants of the coiled-coil domain of IRAG2 reveal no augmentative effect on Ca^2+^ release, suggesting that IRAG2 directly increases the Ca^2+^ release activity of the IP_3_ receptors. Interestingly, IRAG2 shows an augmentative effect on Ca^2+^ release in every IP_3_ receptor subtype; however, it modulates the activity of each IP_3_ receptor subtype in a subtly different manner [83].

In murine platelets, our preliminary data show an augmentative effect of IRAG2 on Ca^2+^ release after stimulation with the agonists thrombin and collagen, which induce platelet aggregation [84]. Moreover, we detected an enhancement of basal Ca^2+^ release through IRAG2 in pancreatic acinar cells, which also impacts basal exocrine amylase secretion. Furthermore, pancreatic acinar cells lacking IRAG2 reveal an increased frequency of Ca^2+^ oscillations, suggesting a modulation of the IP_3_ receptor activity through IRAG2 [9]. However, the mechanism of how IRAG2 modulates Ca^2+^ oscillations through IP_3_ receptors and which IP_3_ receptor subtype is responsible for the IRAG2-derived oscillation pattern remains unclear and will be of interest in future investigations.

Recently, Kozono et al. stated that the cleavage of the C-terminal luminal domain of IRAG2 enhances the augmentative effect of IRAG2 on the release of Ca^2+^ from intracellular stores. Mutation of the C-terminus—and therefore mutation of the cleavage site—results in a defect-cleaving event and subsequently to a decreased release of Ca^2+^ via IP_3_ receptors. Therefore, this cleavage event might be a crucial step for the function of IRAG2 as an IP_3_ receptor regulator. However, the molecular mechanisms leading to this effect still remain unclear [62].

**Figure 3:**
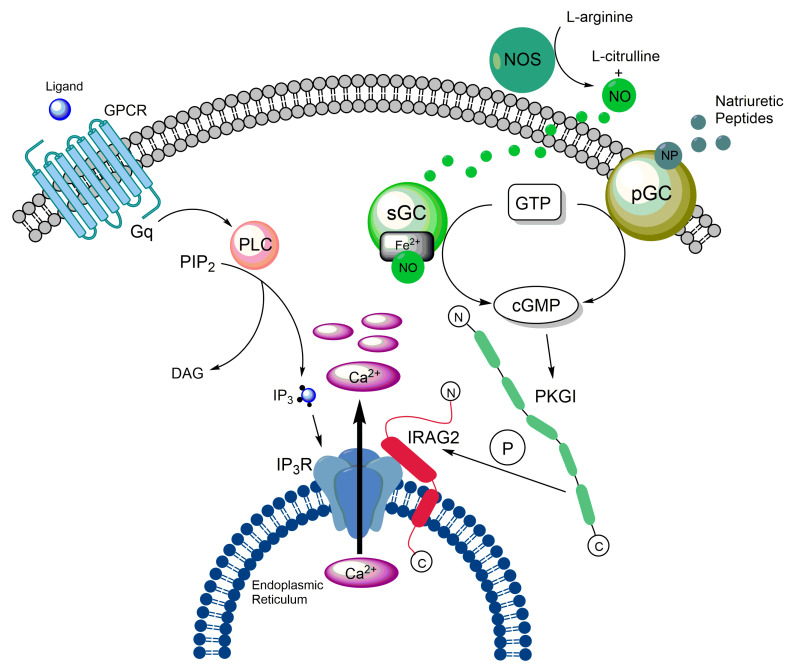
Graphical illustration of the PKGI/IRAG2 signaling pathway. IRAG2 is phosphorylated in a cGMP-dependent manner and enhances Ca^2+^ release from the ER. The enhanced Ca^2+^ release might be mediated due to PKGI-dependent phosphorylation of IRAG2.

Taken together, these data suggest that IRAG2 enhances the release of Ca^2+^ from intracellular stores by regulating the activity of IP_3_ receptors (Figure 3). As IRAG1 inhibits Ca^2+^ release from the ER, IRAG2 might be a counterpart of IRAG1.

### 3.5. IRAG2 as a Substrate of cGMP-Dependent Protein Kinase I

As described in Section 2.1, IRAG1 forms a ternary complex with PKGIβ and IP_3_R-I, in which IRAG1 is phosphorylated by PKGIβ. This phosphorylation results in an inhibition of Ca^2+^ release from the ER [1,2,3,5,36]. The interaction site of IRAG1 with PKGIβ is located between amino acids 152 and 184; however, this site is missing in IRAG2 [1,14]. Furthermore, we could not detect a stable interaction between IRAG2 and PKGIβ in murine platelets [64]. Despite the absence of a stable interaction site with PKGI, a quantitative phosphoproteomics study revealed phosphorylation of human LRMP at amino acids Ser363, threonine (Thr) 375, and Ser418 upon stimulation with NO donors or riociguat in platelets [85]. Remarkably, the phosphorylation sites Ser363 and Thr375 in IRAG2 (consensus sequences: RSAS363 and RRVT375) (Figure 2) and the identified phosphorylation sites Ser664 and Ser677 in human IRAG1 (consensus sequences: RSMS664 and RRVS677) are very homologous. Moreover, we detected cGMP-dependent phosphorylation of IRAG2 in murine platelets [64]. As cGMP predominantly activates PKGI [35,86], we assume that phosphorylation of IRAG2 after cGMP-stimulation is achieved through PKGI (Figure 3). However, it still needs to be investigated which isoform of PKGI—PKGIα and/or PKGIβ—is responsible for the phosphorylation of IRAG2. As murine platelets predominantly express PKGIβ and human platelets only express PKGIβ, IRAG2, like IRAG1, might be a substrate of PKGIβ [5].

### 3.6. (Patho-)Physiological Functions of IRAG2

#### 3.6.1. Function of IRAG2 in Intestinal Type 2 Immunity

The tumor suppressor p53 is crucial for the function of intestinal tuft cells to trigger type 2 immune response after parasitic infections. Previously, it was described that this regulation is mediated by IRAG2. Expression of IRAG2 is transcriptionally regulated by p53, where p53 ensures a high expression of IRAG2 [63]. Furthermore, IRAG2 is expressed in intestinal tuft cells and interacts with the IP_3_R-II in MEF cells [63], which is the major IP_3_ receptor subtype in intestinal tuft cells [87]. A deficiency of p53 leads to lower levels of IRAG2 and results in an impaired Ca^2+^ flux [63]. However, Ca^2+^ flux in intestinal tuft cells is critical for the release of the cytokine IL-25, which triggers the type 2 immune response [87]. Taken together, it is suggested that p53 leads to a high expression of IRAG2 in tuft cells, which ensures Ca^2+^ flux in these cells and, consequently, the release of IL-25. In turn, the release of IL-25 is necessary for type 2 immune response upon parasitic infections. Further, it is stated that the knockdown of IRAG2 results in a higher amount of parasites in the feces of *Irag2*-deficient mice, which might be a result of the impaired type 2 immune response [63].

#### 3.6.2. Function of IRAG2 on HCN Channels

Contrary effects of IRAG1 and IRAG2 on the function of ion channels were already described by Peters et al. [18]. As stated in Section 2.1 and Section 2.4.2, HCN4 channels are regulated by IRAG1. These channels are activated in a voltage-dependent manner and are modulated by cAMP. Binding of cAMP results in a depolarizing shift, leading to an increased opening of the channels [88,89,90]. IRAG1 reveals a function on HCN4 channels by shifting the voltage-dependent activation to more depolarized potentials in the absence of cAMP, resulting in an enhanced opening of the channels. Besides the interaction of HCN4 channels with IRAG1, the interaction of IRAG2 with HCN4 channels was observed, too. However, IRAG2 does not lead to a gain of function, but causes a loss of function by reducing the cAMP-dependent shift of HCN4 channels to more depolarizing potentials. As IRAG1 and IRAG2 are both expressed in sinoatrial nodes, these data suggest important roles for both proteins in the regulation of cellular excitability. Thereby it is suggested that IRAG2 limits the increase in I_f_ in response to stimulation of β-adrenoceptors [18,34]. Furthermore, these data contribute to our thesis that IRAG2 might be a counterpart to IRAG1.

#### 3.6.3. Potential Role of IRAG2 in Taste-Signal Transduction

Expression of IRAG2 was shown in sweet, bitter, and umami taste receptor-expressing cells of murine circumvallate, foliate, and fungiform papillae. In the circumvallate papillae, coexpression with the IP_3_R-III was detected [8]. Moreover, the direct interaction of IRAG2 and IP_3_R-III was shown in several cell lines and tissues [8,9,64,83]. The IP_3_/Ca^2+^ signal cascade plays a significant role in sweet, bitter, and umami taste signal transduction. Activation of phospholipase Cβ_2_ (PLCβ_2_) in these cells results in the production of IP_3_, which induces Ca^2+^ release through IP_3_R-III. This IP_3_-mediated Ca^2+^ release is necessary for the transduction of taste signals. Mice lacking PLCβ_2_ or IP_3_R-III exert a deficiency in their ability to detect sweet, bitter, and umami taste substances [91,92]. Consequently, the interaction of IRAG2 with IP_3_R-III suggests a role for IRAG2 in taste signal transduction [8]. However, to examine the exact function of IRAG2 in taste cells, further experiments are required.

#### 3.6.4. Function of IRAG2 in Exocrine Pancreatic Acinar Cells

Recently we reported the expression of IRAG2 in exocrine pancreatic acinar cells [9]. As IRAG2 interacts with all subtypes of IP_3_ receptors in the pancreas and enhances basal Ca^2+^ release in isolated pancreatic acinar cells (s. 3.4.), we investigated the impact of this effect on the physiological functions of the exocrine pancreas [9]. The main task of pancreatic acinar cells is the secretion of pancreatic juice, consisting of different enzymes, which are needed for the digestion of food components. The release of Ca^2+^ and the secretion of digestive enzymes, such as amylase, are strongly linked. An increase in Ca^2+^ release triggers the secretion of amylase from granules [93,94,95]. In concordance with this, we found that the increased basal release of Ca^2+^ due to IRAG2 also leads to an increase in basal amylase secretion. Mice lacking IRAG2 reveal a decreased basal Ca^2+^ release and, therefore, also a reduced basal secretion of amylase. However, in turn, this does not seem to affect nutrient digestion dramatically, as *Irag2*-deficient animals show no significant differences in body weight compared to wild-type animals [9]. Further, it remains unclear how IRAG2 affects other digestive enzymes, such as lipase or trypsin, as well as bicarbonate production and secretion, which requires further investigation. Immunohistochemical studies also showed a higher amount of amylase in the pancreatic acinar cells of *Irag2*-deficient mice compared to wild-type pancreatic acinar cells. As premature activation of digestive enzymes in the acinar cells contributes to the emergence of acute pancreatitis [95], IRAG2 might also reveal a protective effect against pancreatic diseases, such as acute pancreatitis. However, this topic must be investigated in future experiments using mouse models of acute pancreatitis.

#### 3.6.5. Function of IRAG2 in Platelets

Platelet activation and aggregation upon vascular injury is essential for primary hemostasis, but is also involved in the formation of occlusive thrombi [96]. IRAG1 is phosphorylated by PKGIβ, which causes an inhibition of IP_3_ receptor-mediated Ca^2+^ release from the ER [1,2]. In turn, this results in a reduced aggregability of platelets and also prevents the formation of occlusive thrombi [5,36]. We previously reported the expression of IRAG2 in platelets, where it interacts with all subtypes of IP_3_ receptors [64]. Interestingly, in contrast to IRAG1, our preliminary data show that IRAG2 enhances IP_3_ receptor-mediated Ca^2+^ release from the ER in murine platelets [84]. Moreover, we found that this enhanced Ca^2+^ release increases the aggregation rate of platelets. Platelets lacking IRAG2 reveal reduced Ca^2+^ release and platelet aggregation [64,84]. This effect seems to be mediated by phosphorylation of IRAG2 upon stimulation with cGMP-analogues, as *Irag2*-deficient animals show an even more reduced platelet aggregation after stimulation with cGMP or the NO-donor sodium nitroprusside [64]. Consequently, our results suggest that PKGI-dependent phosphorylation of IRAG2 in platelets causes an increase in Ca^2+^ release, which results in enhanced platelet aggregation (Figure 3). This contrasts with the effect of IRAG1 in platelets. Therefore, we assume that IRAG1 and IRAG2 are counterparts in platelets. Furthermore, as IRAG2 shows an augmentative effect on Ca^2+^ release, it might also contribute to the formation of occlusive thrombi, which can cause severe cardiovascular diseases. Therefore, IRAG2 might be a potential target for the prevention of those diseases.

### 3.7. Significance of IRAG2 as a Prognostic Marker of Cancer

As described in Section 3.2, expression of IRAG2 was shown in a variety of lymphoid tissues and cell lines, such as germinal center (GC) B-cells [10]. Lymphochip array studies revealed that IRAG2 is one of the GC genes whose overexpression defines the good-prognosis GC subcategory of diffuse large B-cell lymphoma (DLBCL) [97,98]. Additionally, in an RT-PCR-based study, high expression of IRAG2 was associated with a good prognosis of DLBCL [99]. It is shown that IRAG2 is involved in the delivery of peptides to MHC class I molecules in a TAP-independent manner [100]. This could be of interest for the behavior of DLBCL upon IRAG2 overexpression, as there is evidence that loss of expression of MHC class II molecules is associated with a poor outcome. Additionally, MHC class II-negative cases show a decrease in tumor-infiltrating CD8-positive T-cells [101,102]. This might explain the association between IRAG2 overexpression and a good prognosis of DLBCL [10].

In breast cancer, a correlation is seen between disease-free survival and the expression of genes that participate in immune response regulation and the normal development of lymphoid tissues. One of these genes is the *IRAG2* gene. Therefore, high expression of *IRAG2* might be a promising biomarker of life expectancy [103].

Preliminary studies also showed the expression of *IRAG2* in some cases of ovarian cancer, which suggests that *IRAG2* may represent a potential marker in the field of ovarian cancer [10]. However, further investigations are therefore needed.

Recently, IRAG2 expression was examined in lung adenocarcinoma. Thereby, it is shown that the IRAG2 gene and protein expression is lower in patients with lung adenocarcinoma as well as in lung adenocarcinoma cell lines. Additionally, high expression of *IRAG2* correlates with a better prognosis of patients, indicating that IRAG2 is a positive prognostic predictor. In vitro experiments demonstrated that overexpression of the IRAG2 protein could decrease the proliferation, migration, and invasion in A549-cells. Furthermore, multiple oncogenic signaling pathways, e.g., the p-STAT3, p-PI3K-p-AKT, p-MEK, and EMT pathways, are downregulated when *IRAG2* expression is enhanced. *IRAG2* is also positively associated with various tumor-infiltrating immune cells and their markers. These data suggest that *IRAG2* might function as a tumor suppressor gene. Additionally, high *IRAG2* expression correlated with an upregulation of immune checkpoints. Hence, IRAG2 could also be involved in the immunotherapy response of lung adenocarcinoma patients using checkpoint inhibitors [104].

Lower *IRAG2* expression was detected in many other solid cancers, e.g., breast invasive carcinoma, colon adenocarcinoma, glioblastoma multiforme, kidney chromophobe, kidney renal papillary cell carcinoma, prostate adenocarcinoma, rectum adenocarcinoma, and uterine corpus endometrial carcinoma [104]. This suggests a role for IRAG2 overexpression also in other solid cancers.

IRAG2 seems to be involved in the cellular response following boron neutron capture therapy (BNCR). In this therapy, ^10^B-boronophenylalanine can be preferentially taken up into tumor cells. In combination with radiation, this therapy results in the apoptosis of the tumor cells. Sato et al. described that a higher amount of fragmented IRAG2 is generated in human squamous carcinoma in response to BNCR. Hence, fragmentation of IRAG2 could be involved in the cellular response of BNCR and, therefore, in BNCR-induced cell death. Additionally, IRAG2 fragmentation could be considered a potential biomarker for cell damage in response to BNCR. However, further investigations are needed to evaluate this potential role [105].

### 3.8. Significance of IRAG2 Polymorphisms

The pulmonary adenoma susceptibility 1 (*Pas1*) locus plays a major role in the predisposition of lung tumors [106]. The *IRAG2* gene is localized to this locus. Furthermore, expression of *IRAG2* was detected in normal human lung tissue and human lung adenocarcinomas, as well as in mouse normal lung tissue and mouse lung tumors. Thereby, no obvious differences between normal lung and lung adenocarcinoma tissue in *IRAG2* expression levels were observed [107,108,109]. This, however, is in contrast to the data of Jin et al., who detected a lower IRAG2 gene and protein expression in lung adenocarcinoma tissue [104]. It is also shown that an *IRAG2* Pro537Leu nonconservative variation was linked with an inflammatory response in the lungs of several mouse strains [110]. Furthermore, two SNPs were found for human *IRAG2*: a Val141Leu variation and a Ser197Cys variation. In animal studies, *IRAG2* polymorphisms increased the risk of lung tumors. In humans, the analysis revealed no significant association of these SNPs with enhanced lung cancer risk [104,107,108,109]. However, in patients with age at tumor onset ≤65 years, survival rates of patients carrying either the Leu/Leu or the Val/Leu genotype differed significantly from those with the Val/Val genotype. Kaplan–Meier analysis demonstrated that the median follow-up at death was 33 months for Leu allele carriers and 100 months for Val/Val cancer patients. These findings suggest that the *IRAG2* Val141Leu polymorphism can predict survival in lung adenocarcinoma. A hypothesis for the impact of the Val141Leu SNP on the survival rate might be an altered modulation of patients’ immune systems [109], as it was recently suggested that IRAG2 might act as a tumor suppressor that is involved in the progression of lung adenocarcinoma (s. 3.7.) [104]. The Ser197Cys SNP, however, showed no effect on the survival of lung cancer patients [109].

The *IRAG2* gene is localized at the insulin-dependent diabetes (idd) susceptibility locus (idd6). A high number of SNPs were found on this locus that were associated with type 1 diabetes. As idd6 seems to be involved in the control of T-cell survival and proliferation, candidate genes for idd6 are those that are implicated in the immune system [12]. Therefore, *IRAG2* might be a strong candidate for this locus, as it is expressed in a variety of lymphoid tissues and cell lines, such as B-and T-cell lines [7,12]. Additionally, a high number of *IRAG2* SNPs is found on this locus in non-obese-diabetes (NOD) mice, which contributes to the thesis that *IRAG2* might have an impact on the development of diabetes [12].

Additionally, it is reported that the idd6 diabetes susceptibility region controls defective expression of the *IRAG2* gene in NOD mice, where the NOD allele at this locus mediates lower mRNA expression levels of *IRAG2*. This leads to the hypothesis that decreased expression of *IRAG2* in these NOD mice might constitute a type 1 diabetes susceptibility factor in this Idd6 region [11].

## 4. Conclusions and Outlook

As depicted in this review, IRAG1 and IRAG2 are membrane proteins that regulate intracellular Ca^2+^ (Table 1) and are substrate proteins of PKGI. IRAG1 is located at the ER [2,6], whereas IRAG2 was not only found at the ER, but also at other cellular localizations, such as the outer nuclear membrane [60] or the Golgi apparatus [81]. Hence, it can be speculated whether IRAG1 might be localized at further intracellular positions and if it exerts further, and up to now not established, functions there. In this regard, it is interesting that IRAG1 and IRAG2 are modulators of HCN4 channels that are located at the plasma membrane [34] (Table 1). Hence, IRAG1 and IRAG2 might also be found at the plasma membrane or other locations where the ER plasma membrane is associated.

Until now, the regulation of the IP_3_R by IRAG1 and IRAG2 is not fully understood at the molecular level. Therefore, it would be tempting to reveal the molecular mechanism by which the PKGI-mediated phosphorylation of IRAG1 inhibits and of IRAG2 activates the intracellular Ca^2+^ via the IP_3_R. These mechanisms might also point to new target sites for the development of novel pharmacological modulators of IP_3_R. Modulators of the IP_3_R could lead to valuable pharmacological drugs for various diseases, e.g., in the gastrointestinal tract, in the lung, in the heart, in immune cells, or in cancer cells.

The functional interplay of IRAG1 and IRAG2 in tissues and cells is a topic that needs further exploration. Several studies indicate that IRAG1 and IRAG2 function as functional counterparts in platelets or in pacemaker cells. However, this regulation might differ in diverse other cells and might depend on other factors, e.g., on PKG-mediated phosphorylation of IRAG1 and/or IRAG2, and protein splice variants [21] on other protein modifications, e.g., glycosylation or truncation.

IRAG1 and IRAG2 could serve as biomarkers for several diseases in the future. Upregulated expression of IRAG1 was associated with a bad prognosis of solid tumors [40,44], whereas the high expression level of IRAG2 could be a beneficial marker for the prognosis of lung tumors or the development of type 1 diabetes. Several polymorphisms were elucidated for IRAG1 or IRAG2. For IRAG1, there were polymorphisms identified which are associated with migraine and CeAD, with achalasia and moyamoya syndrome, asthma, and arterial thrombosis. For IRAG2, polymorphisms are coupled to the risk of cancer diseases, e.g., lung tumors, diabetes, or immune diseases. Hence, these polymorphisms could serve as prognostic markers for these diseases. Regarding tumor diseases, it is noteworthy that both IRAG1 and IRAG2 were reported to possibly act as tumor suppressor genes [6,104]. However, the mechanism regarding this aspect was not elucidated up to now and would be tempting to determine in the future.

Taken together, these findings indicate that IRAG1 and IRAG2 might be applicable as potential targets for therapeutical and diagnostic biomarker/polymorphism approaches, e.g., in diverse cardiovascular, gastrointestinal, and cancer diseases. Further molecular and functional research will be needed to exploit these different prospects.

## Figures and Tables

**Table 1:** Effects of IRAG1 or IRAG2 protein interactions.

	Function by Interaction with Proteins	References
**IRAG1**	**IP_3_R/PKGIβ:**	
	cGMP- and cCMP-dependent inhibition of Ca^2+^ signaling (smooth muscle and platelets)	[1,2,3,4,5,13,21,22,30,36]
	**TRPM4:**	
	NO/cGMP-dependent modulation of TRPM4 activity	[20]
	**HCN4:**	
	enhanced opening of the channels	[18,34]
**IRAG2**	**IP_3_R:**	
	activation of Ca^2+^ signaling (platelets, pancreas, and MEF and HEK cells)	[9,63,64,83,84]
	**HCN4:**	
	reduced opening of the channels	[18,34]
	**SUN proteins:**	
	IRAG2 takes part in LINC complex as KASH protein: microtubule interaction and maintaining nuclear shape	[60]

## Data Availability

Requests to access the figures should be directed to the corresponding author: Jens.Schlossmann@chemie.uni-regensburg.de.
